# Estimation of ground reaction force waveforms during fixed pace running outside the laboratory

**DOI:** 10.3389/fspor.2023.974186

**Published:** 2023-02-13

**Authors:** Seth R. Donahue, Michael E. Hahn

**Affiliations:** Bowerman Sports Science Center, Department of Human Physiology, University of Oregon, Eugene, OR, United States

**Keywords:** biomechanics, sensors, kinetics, running, machine learning, inertial measure unit

## Abstract

In laboratory experiments, biomechanical data collections with wearable technologies and machine learning have been promising. Despite the development of lightweight portable sensors and algorithms for the identification of gait events and estimation of kinetic waveforms, machine learning models have yet to be used to full potential. We propose the use of a Long Short Term Memory network to map inertial data to ground reaction force data gathered in a semi-uncontrolled environment. Fifteen healthy runners were recruited for this study, with varied running experience: novice to highly trained runners (<15 min 5 km race), and ages ranging from 18 to 64 years old. Force sensing insoles were used to measure normal foot-shoe forces, providing the standard for identification of gait events and measurement of kinetic waveforms. Three inertial measurement units (IMUs) were mounted to each participant, two bilaterally on the dorsal aspect of the foot and one clipped to the back of each participant's waistband, approximating their sacrum. Data input into the Long Short Term Memory network were from the three IMUs and output were estimated kinetic waveforms, compared against the standard of the force sensing insoles. The range of RMSE for each stance phase was from 0.189–0.288 BW, which is similar to multiple previous studies. Estimation of foot contact had an *r*^2^ = 0.795. Estimation of kinetic variables varied, with peak force presenting the best output with an *r*^2^ = 0.614. In conclusion, we have shown that at controlled paces over level ground a Long Short Term Memory network can estimate 4 s temporal windows of ground reaction force data across a range of running speeds.

## Introduction

Wearable sensors are being used extensively for the collection of human running biomechanical data outside of the laboratory (1–5). The primary wearable sensors used recently in locomotion biomechanics have been multi-axial inertial measurement units (IMUs), which measure linear acceleration, angular velocity data as well as the local magnetic field. Previously, IMUs have been used to estimate gait events, contact time, and other spatial temporal variables (2, 6–8). Additionally, specific kinetic variables have been estimated from IMU data collected in laboratory settings, such as joint moments, peak vertical force, impulse and loading rate (1, 6, 9, 10). Other wearable sensors utilized for biomechanical monitoring or clinical evaluation are insole force sensors. These sensors measure force between the foot and shoe, and have been validated as a measure of vertical ground reaction forces (GRF) during locomotion on a treadmill (11). Wearable sensors have the capability to also be incorporated into other sensor systems for the estimation of specific external loading variables and internal tissue loading (12, 13), and for overall feedback during training (14, 15).

Previous studies have reported either statistical or physics based models for the estimation of kinetic variables associated with external loading (6, 16, 17). In recent years, machine learning techniques have been used instead of physical or statistical modelling, having become a popular and robust set of tools for biomechanical analysis and estimation of kinematic and kinetic variables without having a precise mathematical description of the underlying mechanics. Previous work using machine learning algorithms have estimated or predicted gait events from IMU data (18–21); with IMUs located either bilaterally on the feet or on the sacrum. Machine learning algorithms, including artificial neural networks (ANNs), recurrent neural nets (RNNs), among other techniques, have also been used to estimate kinetic variables, such as vertical impulse, loading rate and peak GRFs (9, 10, 22–24). These previous methods focused on feature engineering, and the extraction of specific features to make estimations from various waveforms to inform estimation. A few drawbacks for these studies include the biomechanical expertise required for estimation of these variables, significant preprocessing of raw data, and identification of stance phase before data can be parsed into a usable form. One of the advantages of modern machine learning algorithms such as the Long Short Term Memory network (LSTM), is the ability to extract meaningful features from a given time series data. Essentially, this presents as a machine translation problem, translating IMU data to kinetic data. Furthermore, these machine learning models are yet to be tested on data collected outside the laboratory, as they have been built using data from controlled laboratory settings and do not capture the variability of human movement that occurs out of the laboratory in response to surface differences and changes in velocity (25).

The present work is the next step in the development of modeling techniques for the estimation of kinetic variables outside of the laboratory and testing the performance of these models using Leave One Out Cross Validation (LOOCV). We propose the use of Long Short Term Memory networks (LSTMs) to map IMU data onto GRF waveforms measured with force sensing insoles from participants running on a track at a set pace. The LSTM approach was specifically developed for time series data, and mapping between two different waveforms (26). The sequence-to-sequence regression allows the LSTM to identify features and estimate the GRF waveform in a manner that requires no feature engineering. The purpose of this study was to implement a machine learning algorithm for the mapping of inertial data to kinetic waveforms from participants running on a track across a range of velocities.

## Methods

This study was approved by the University of Oregon Institutional Review Board (protocol # 10062020.007). Data were collected from 15 participants (Table 1), (9 male, 6 female, age: 23.6 years, height: 178.3 cm, mass: 73.5 kg) as part of a larger, ongoing project. All analyses were performed in custom Matlab programs (Mathworks, Natick, MA). Multi-axial IMUs (Casio, Tokyo, Japan) were mounted bilaterally on the dorsal aspect of each participants feet and approximately on the sacrum (clipped on the back of each participant's waistband). These sensors recorded 3D linear accelerations and angular velocities at 200 Hz (acceleration measurement range 0–16 g). The use of multiple inertial sensors has been suggested to lead to improved estimation of spatial temporal and kinetic variables, compared to a single inertial sensor (6, 27, 28). Foot-shoe normal force data were recorded with Loadsol insole force sensors bilaterally in between the foot and the shoe (Novel Electronics, St. Paul, MN) at 100 Hz. The force sensing insoles and IMUs were synced using foot-stomps before each trial. Based upon their 5-km race pace participants performed a total of 4 or 5 paces on a 400 m square track, with the fastest pace being optional. Each participant monitored their pace with Garmin GPS, (Kansas City, KS). If they missed their expected time trial duration by more than 2 s, they would be asked to repeat the trial, after sufficient rest. An exemplar set of paces is shown in Table 2. The total range of velocities run by participants in this study was 2.33–5.36 m s^−1^. These velocities represent typical training and race paces for the majority of recreational and high-level distance runners (6, 29).

**Table 1:** Participant demographics.

Sex	Age (years)	Mass (kg)	Height (cm)	Pace range (m s^−1^)
M	59	68	180	2.68–3.35
M	20	79	180	2.55–3.16
M	18	82	183	3.83–4.88
M	18	79	183	3.83–5.36
M	19	61	173	3.83–5.36
M	20	68	173	3.83–5.36
M	20	67	178	3.58–4.47
M	29	70	185	3.83–5.36
M	34	68	180	3.35–4.47
F	19	68	170	2.33–2.82
F	20	66	170	2.98–3.83
F	20	66	170	2.98–3.83
F	20	63	168	3.16–4.13
F	20	63	168	3.35–4.47
F	20	54	173	3.58–4.88

**Table 2:** Example paces for 400 m run.

Example Paces	Minutes per mile	Average velocity (m s^−1^)
Pace 1	8:30	3.16
Pace 2	8:00	3.35
Pace 3	7:30	3.57
Pace 4	7:00	3.83
Pace 5 (Optional)	6:30	4.12

## Data processing

**Figure 1:**
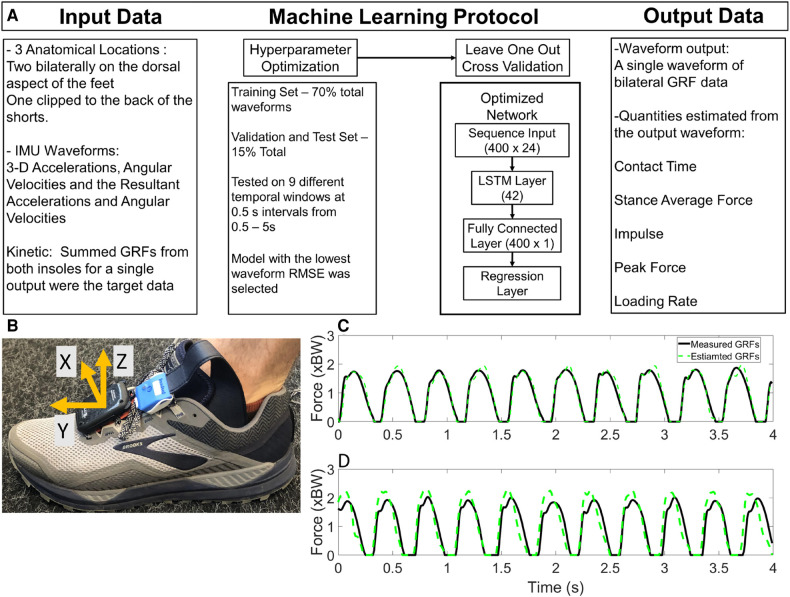
Panel **A**, machine learning methodology and throughput, specifically the input data, machine learning protocol and output. Calculated output contact time and kinetic variables are shown here. Panel **B** instrumentation on the foot, with the Kalman corrected coordinate system on the IMU. The Sacral IMU was positioned with the orange clip to the posterior of the participant, so the IMU proper was between the participants skin and the shorts to minimize the movement of the sensor. Panel **C** measured and estimated ground reaction forces from a participant with the smallest RMSE for a participant, Panel **D** shows the largest RMSE measured and estimated ground reaction forces from the same participant.

Data from the IMUs were post processed with a Kalman filter to orient the local (IMU) coordinate system vertical to gravity prior to any post processing in Matlab (Figure 1 Panel B). The IMU data were down sampled to 100 Hz to match the force sensing insole sampling frequency and then filtered with a 4th order low-pass zero-lag Butterworth filter (*fc *= 35 Hz). Foot-shoe normal force data were measured from force sensing insoles, considered the standard reference for identification of measured gait events and kinetic variables in this study (11). Kinetic data were filtered with a 2nd order low-pass zero-lag Butterworth filter (*fc *= 20 Hz). Internal clock drift between the insoles and the IMUs was rectified by an iterative corrections algorithm. This algorithm adjusted the IMU and kinetic data such that it approximately matched each IC from both systems within ±0.02 s, by removing or adding zeros in the previous swing phase of the kinetic data. The identification of IC from the IMU data is detailed in previous work from our laboratory (30). Force data <5% body weight (BW) were set to 0 BW. The estimated kinetic waveforms output from the machine learning algorithm were filtered with the same filter as the measured kinetic waveforms. Estimated foot ground contacts less than 0.050 s were set to 0 BW, as foot contacts shorter than 0.050 s were considered noise, having small magnitudes and not consistent with measured foot contacts observed in running locomotion.

### Machine learning architecture

The overall structure of the machine learning algorithm is shown in (Figure 1 Panel A). Briefly, the sequence input was 400 × 24 for the input channels (acceleration, angular velocity and their resultant magnitudes), and the output was a 400 × 1 GRF waveform (sum of the left and right GRF waveform data from the force sensing insoles). The activation functions of the LSTM are described elsewhere (26, 31). The steps for development and testing of the machine learning models were two-fold; first the network architecture was optimized using the Bayesian Optimization for Deep Learning (32), and then the network was tested using Leave One Out Cross Validation (LOOCV). Bayesian Optimization for Deep Learning requires user specification of the hyperparameters, which are then optimized. The Bayesian Optimization was conducted on the data set with 70% Training, 15% Validation and 15% Test segmentation of the data (Figure 1). The optimal network architecture was determined by performance on the test data set. The initial set of hyperparameters optimized included the initial learning rate, gradient decay factor, squared gradient decay factor, L2Regularization and number of hidden units. From the hyperparameter optimization only the number of hidden units were observed to influence the outcome of the machine learning protocol. All other hyperparameters were therefore set to the recommended values from MATLAB for sequence-to-sequence regression. The range of the number of hidden units used in the optimization was [10–50]. Through the Bayesian Optimization process, the optimal number of hidden units was determined to be 42. This value was used for the LOOCV process. Throughout the hyperparameter optimization process, we tested the range of temporal input data from 0.5 s to 5 s windows. Based on the performance of these optimized algorithms, by analysis of whole waveform RMSE it was found that a 4 s temporal window was the most accurate for the estimation of ground reaction force waveforms.

### Data analysis

Estimated waveforms from the LOOCV were concatenated for each trial, analyzed and are presented in this work. This sets a baseline for the use of these machine learning models as each participant was treated as a novel participant. Initial Contact (IC) was identified by the first instance of force >5% BW and toe off (TO) was determined by the last instance of force greater than >5% BW. Contact time was determined by taking the temporal difference between these two discrete events. Stance average GRFs, impulse, peak GRFs, and average loading rate were the kinetic variables calculated in this work, from the estimated force waveforms. Average loading rate was calculated by identifying the impact peak and then averaging the slope in the middle 60% of the region between IC and the impact peak (33).

Pearson correlation coefficients (*r*^2^) were used to compare the estimated force data output from the LSTM to the measured insole force data. Seventy-five trials were used, with fifteen participants running five velocities, and each data point representing a 400 m time trial on a square track. Differences between the model estimated variables and measured waveform variables are presented in both linear regression and Bland-Altman plots with 95% confidence intervals (CIs) and Limits of Agreement (LoA), respectively. A strong correlation was defined as *r*^2^ ≥ 0.8, a moderate correlation as 0.5 ≤ *r*^2^ ≤ 0.8 and a weak correlation as 0.3 ≤ *r*^2^ ≤ 0.5. Differences between measured and estimated gait events are presented as well as root mean square error (RMSE) for each contact time and kinetic variable.

## Results

**Figure 2:**
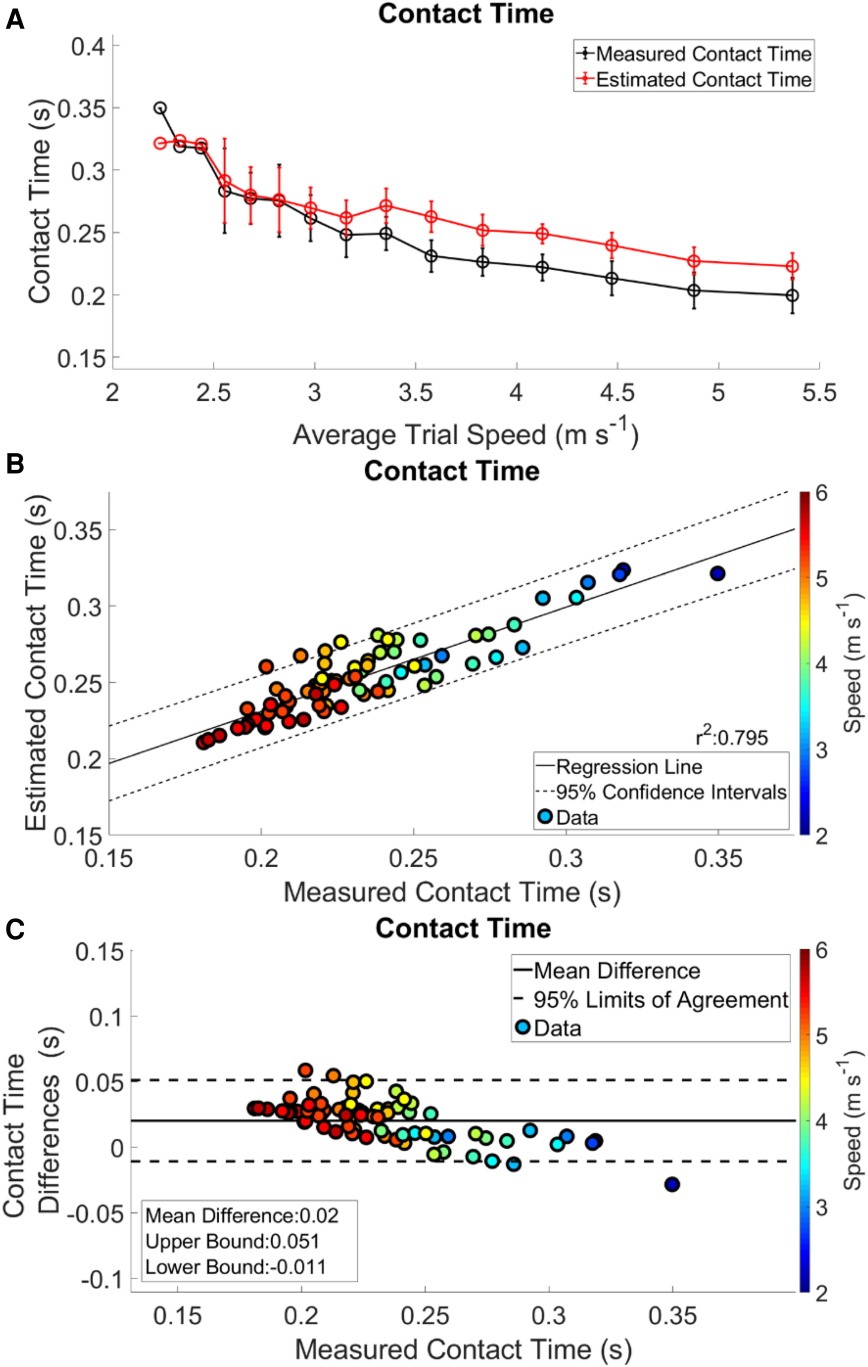
Complete analysis of contact time. Panel **A** shows contact time trends across the range of speeds. The measured contact times are in black and the estimated contact times are in red. Regression analysis and 95% confidence intervals of contact time is in Panel **B**. Panel **C** presents a Bland-Altman plot of the difference between the estimated and measured contact times, and 95% limits of agreement.

The data presented are the trial means from each subject and velocity from the LOOCV analysis. Waveform RMSE ranged from 0.191–0.309 BW, while the individual stance phase RMSE ranged from 0.189 to 0.288 BW (Table 3). Exemplar data for the minimal and maximal RMSE outputs for a participant are shown in (Figure 1 Panel C). Estimated IC was identified prior to measured IC and IC differences ranged from 0.013–0.020 s per trial (Table 4). The identification of TO differences ranged from −0.012 to 0.041 s. At velocities <3.16 m s^−1^, TO was estimated prior to the measured gait event. However, at velocities >3.16 m s^−1^, the estimation of TO occurred after the measured gait event (Table 4). Estimated and measured contact time had good agreement at average running velocities < 3.16 m s^−1^, however at average running velocities >3.16 m s^−1^, contact time was overestimated (Figure 2 Panel A). The Pearson's Correlation Coefficient between the estimated and measured contact time showed a moderate correlation; *r*^2^ = 0.795 (Figure 2 Panel B). Bias in the estimate of contact time was 0.020 with 95% LoA: [−0.011, 0.051] (Figure 2 Panel C). Contact time RMSE ranged from 0.089 s to 0.021 s (Table 5).

**Table 3:** Stance and waveform root mean square error.

Average Speed (m s^−1^)	Stance RMSE (BW)	Waveform RMSE (BW)
2.24	0.230 ± 0.000	0.238 ± 0.025
2.33	0.189 ± 0.000	0.191 ± 0.029
2.44	0.199 ± 0.000	0.202 ± 0.028
2.55	0.253 ± 0.080	0.271 ± 0.100
2.68	0.268 ± 0.056	0.266 ± 0.074
2.82	0.267 ± 0.019	0.274 ± 0.057
2.98	0.281 ± 0.020	0.309 ± 0.059
3.16	0.262 ± 0.038	0.304 ± 0.056
3.35	0.288 ± 0.039	0.305 ± 0.071
3.58	0.248 ± 0.033	0.268 ± 0.062
3.83	0.230 ± 0.049	0.265 ± 0.076
4.13	0.242 ± 0.053	0.281 ± 0.072
4.47	0.233 ± 0.048	0.275 ± 0.073
4.88	0.243 ± 0.043	0.284 ± 0.089
5.36	0.240 ± 0.052	0.287 ± 0.101

**Table 4:** LSTM estimated gait event error.

Average Velocity (m s^−1^)	IC DIFFERENCE (S)	TO DIFFERENCE (S)
2.24	0.013 ± 0.000	0.041 ± 0.000
2.33	0.019 ± 0.000	0.014 ± 0.000
2.44	0.019 ± 0.000	0.015 ± 0.000
2.55	0.015 ± 0.003	0.007 ± 0.003
2.68	0.017 ± 0.005	0.014 ± 0.018
2.82	0.015 ± 0.006	0.015 ± 0.017
2.98	0.015 ± 0.003	0.007 ± 0.017
3.16	0.014 ± 0.006	0.001 ± 0.011
3.35	0.019 ± 0.004	-0.003 ± 0.022
3.58	0.020 ± 0.005	-0.011 ± 0.012
3.83	0.018 ± 0.005	-0.007 ± 0.012
4.13	0.015 ± 0.008	-0.012 ± 0.009
4.47	0.017 ± 0.008	-0.009 ± 0.009
4.88	0.017 ± 0.003	-0.006 ± 0.008
5.36	0.017 ± 0.005	-0.006 ± 0.005

**Table 5:** LSTM estimated spatial-temporal and kinetic variables root mean square error.

Average Velocity (m s^−1^)	Contact time (s)	Stance average (BW)	Impulse (BW*S)	Peak GRF (BW)	Loading rate (BW S^−1^)
2.24	0.090 ± 0.000	0.063 ± 0.000	0.103 ± 0.000	0.266 ± 0.000	13.592 ± 0.000
2.33	0.021 ± 0.000	0.070 ± 0.000	0.032 ± 0.000	0.159 ± 0.000	15.575 ± 0.000
2.44	0.023 ± 0.000	0.070 ± 0.000	0.032 ± 0.000	0.183 ± 0.000	12.559 ± 0.000
2.55	0.028 ± 0.002	0.127 ± 0.082	0.048 ± 0.024	0.227 ± 0.081	9.535 ± 3.032
2.68	0.024 ± 0.001	0.122 ± 0.069	0.041 ± 0.016	0.221 ± 0.053	8.733 ± 1.564
2.82	0.024 ± 0.001	0.108 ± 0.048	0.042 ± 0.012	0.256 ± 0.053	8.695 ± 1.839
2.98	0.025 ± 0.006	0.121 ± 0.037	0.044 ± 0.012	0.208 ± 0.034	7.798 ± 1.242
3.16	0.023 ± 0.006	0.154 ± 0.073	0.041 ± 0.011	0.221 ± 0.018	9.963 ± 4.378
3.35	0.034 ± 0.014	0.150 ± 0.068	0.047 ± 0.013	0.213 ± 0.038	11.383 ± 5.400
3.58	0.039 ± 0.016	0.196 ± 0.091	0.047 ± 0.016	0.225 ± 0.056	14.725 ± 4.691
3.83	0.031 ± 0.012	0.177 ± 0.086	0.037 ± 0.018	0.230 ± 0.107	14.085 ± 3.744
4.13	0.033 ± 0.013	0.196 ± 0.093	0.040 ± 0.022	0.237 ± 0.130	14.825 ± 4.458
4.47	0.032 ± 0.014	0.198 ± 0.093	0.037 ± 0.019	0.226 ± 0.118	15.561 ± 5.319
4.88	0.029 ± 0.008	0.218 ± 0.102	0.034 ± 0.014	0.237 ± 0.118	15.608 ± 3.667
5.36	0.028 ± 0.007	0.198 ± 0.085	0.032 ± 0.014	0.229 ± 0.121	18.132 ± 5.392

**Figure 3:**
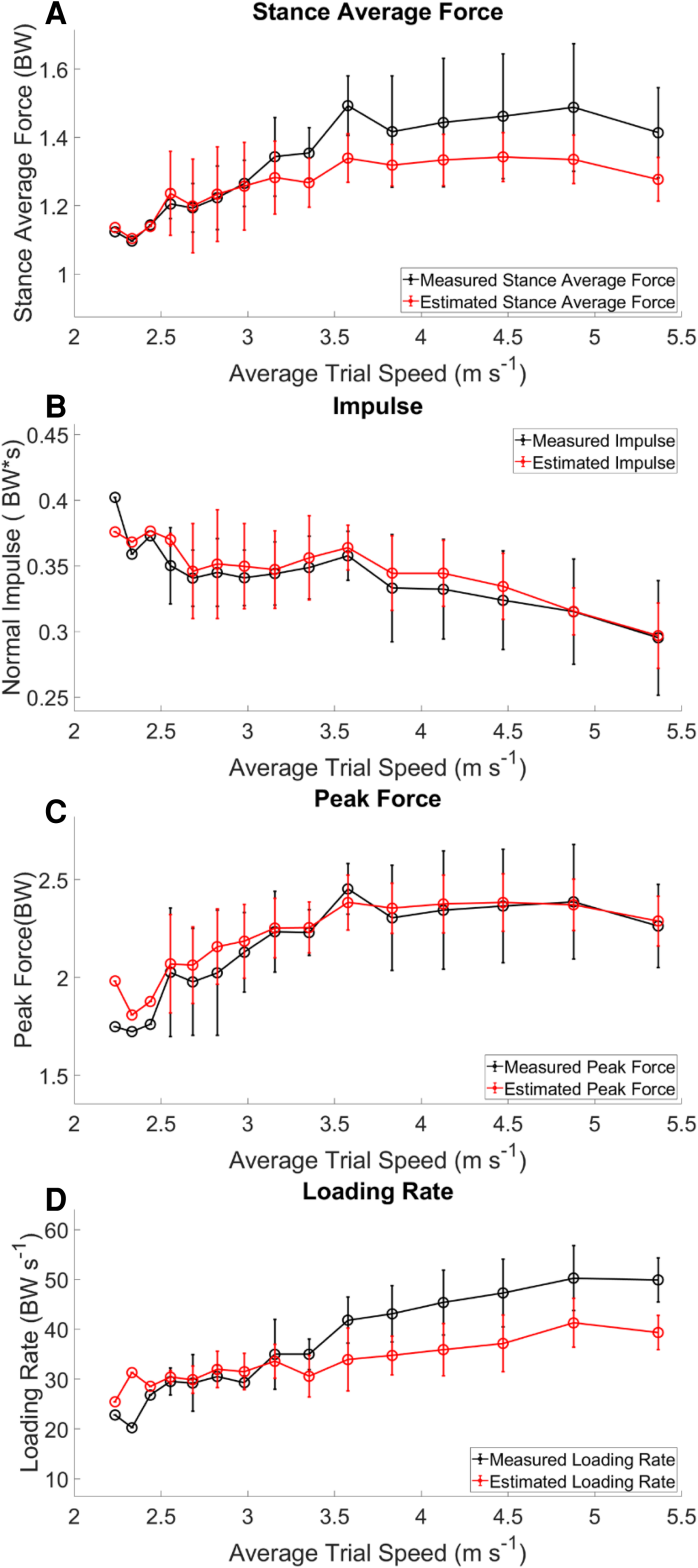
Kinetic variables across velocities. Measured (black) variables calculated directly from the force sensing insoles. Estimated (red) calculated from the LSTM estimated waveform. The trends in the estimated data follow those of the measured data, there is however an offset between the two.

**Figure 4:**
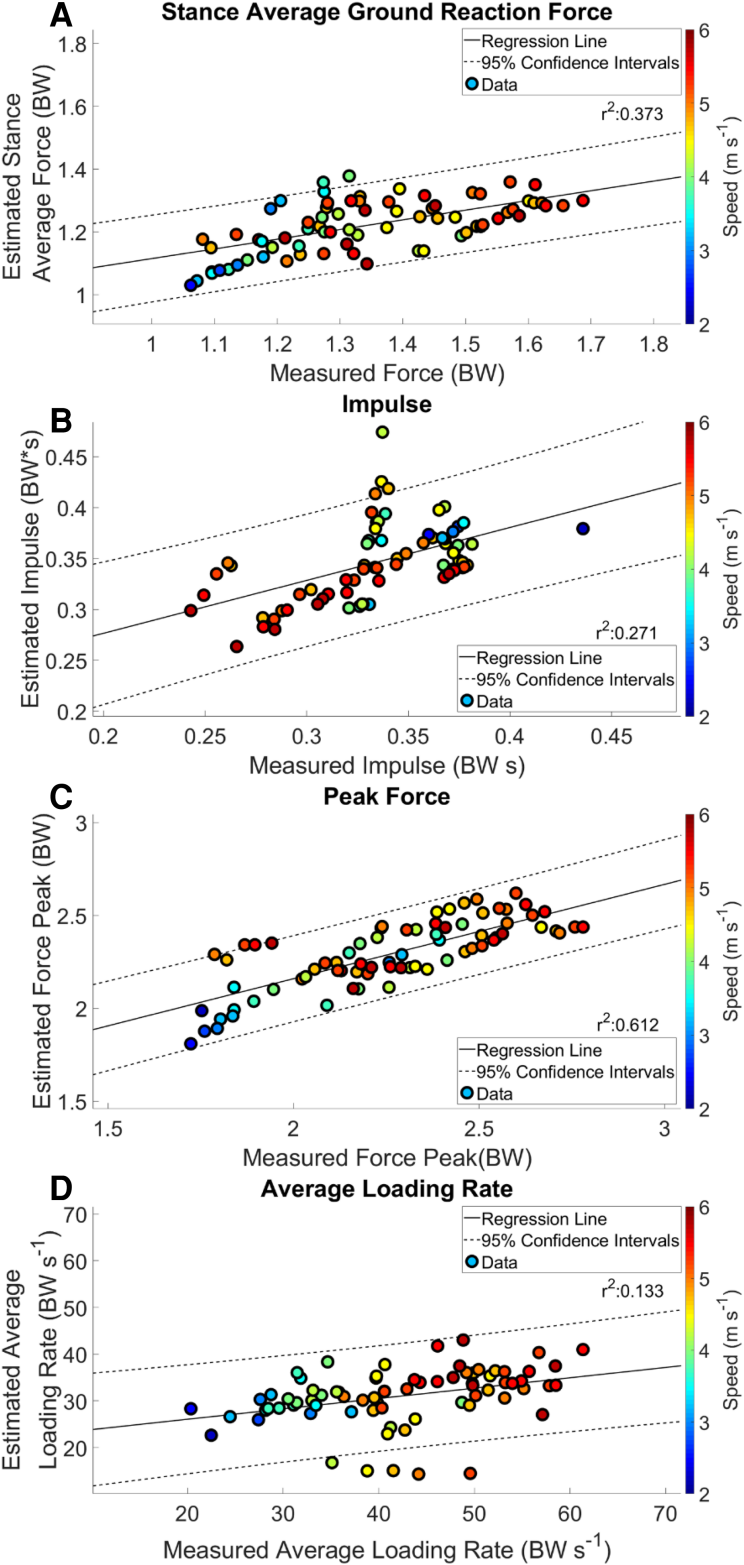
Regression analysis of estimated kinetic variables with mean and 95% confidence intervals. Each data point represents an average speed trial. The color of a trial represents the average running speed of the trial.

**Figure 5:**
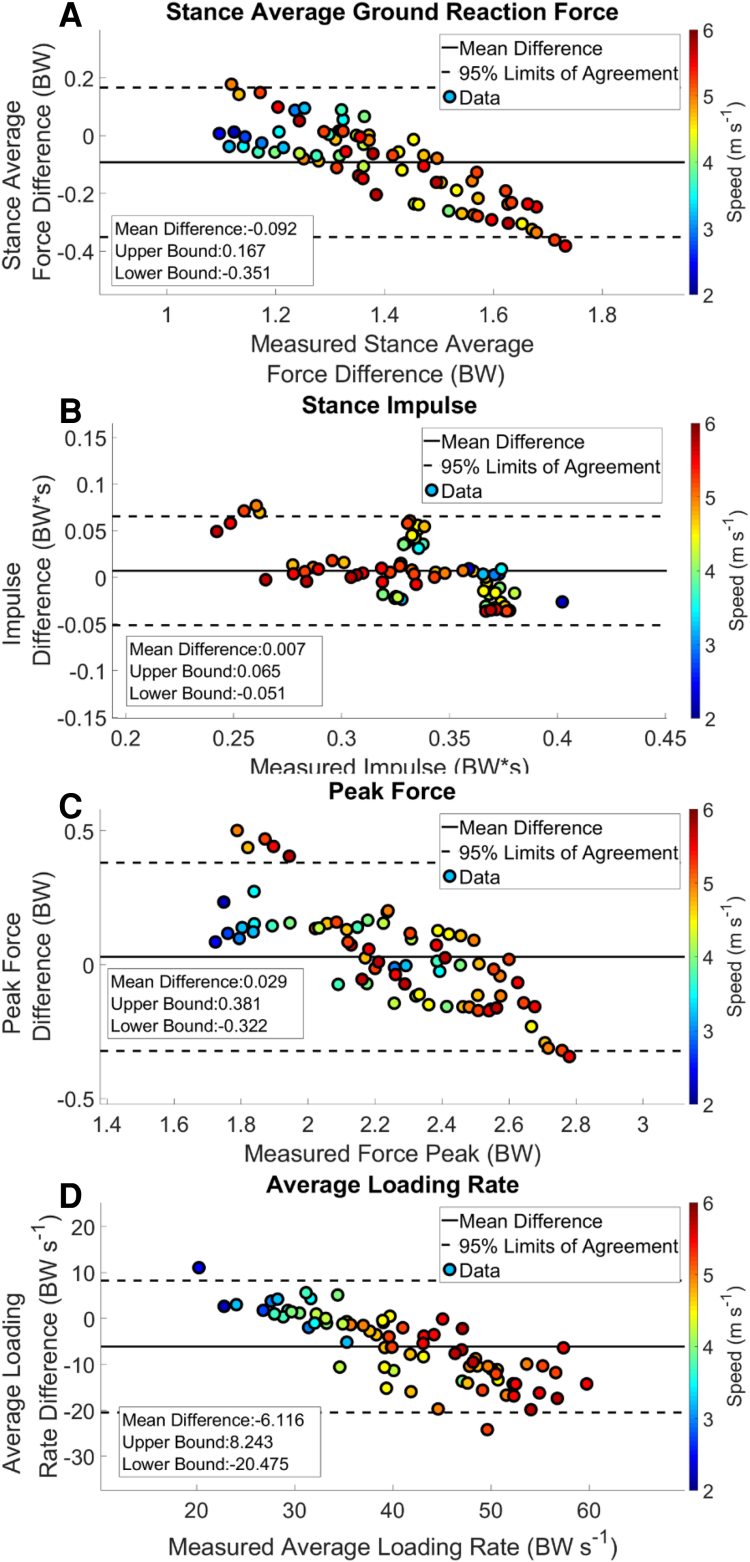
Bland Altman plot with offset and 95% limits of agreement. Each data point represents an average speed trial. The color of a trial represents the average running speed of the trial.

Estimated output from the measured stance average GRFs showed a consistent underestimation at velocities > 3.16 m s^−1^ (Figure 3 Panel A). There was a weak correlation between the estimated stance average GRF and the measured stance average GRF; *r*^2^ = 0.373 (Figure 4 Panel A). The agreement between the estimated stance average GRFs and the measured stance average GRFs were offset by −0.092 BW and 95% LoA [−0.351 0.167] BW (Figure 5 Panel A). The stance average ground reaction force RMSE ranged from 0.063–0.402 BW (Table 5). The measured stance impulse decreased as the average running velocity increased. At all but the slowest velocity (2.24 m s^−1^) and the two fastest velocities (4.88 and 5.36 m s^−1^) the impulse was overestimated by the LSTM output (Figure 3, Panel B). Estimated impulse had a weak correlation with measured impulse; *r*^2^ = 0.271 (Figure 4 Panel B). The agreement between the estimated impulse and the measured impulse bias was 0.007 BW*s and 95% LoA [−0.051 0.065] BW*s (Figure 5 Panel B). The RMSE across the range of velocities ranged from 0.159 to 0.266 BW*s (Table 5).

The estimated peak forces across the range of velocities were similar to the measured peak forces, except for at the slowest velocity (2.24 m s^−1^) (Figure 3 Panel C). Estimated peak GRFs were moderately correlated with the measured peak ground reactions forces (*r*^2^ = 0.612) (Figure 4 Panel C). The agreement between the measured and estimated peak GRFs had an offset of 0.029 BW with 95% LoA [−0.322 0.381] BW (Figure 5 Panel C). The average RMSE of peak vertical GRFs ranged from [0.07 0.218] (BW) (Table 5). The estimated average force loading rate was overestimated compared to measured loading rate across the range of velocities. Estimated loading rate was weakly correlated with measured loading rate (*r*^2^ = 0.133) (Figure 4 Panel D). The agreement between the measured and estimated loading rate had an offset of −6.116 BW s^−1^, with LoA [−20.475 8.243] BW s^−1^ (Figure 5 Panel D). The average RMSE for loading rate over each velocity ranged from [7.798 to 18.132] BW s^−1^ (Table 5).

## Discussion

The purpose of this study was to implement a machine learning algorithm for the mapping of **IMU** data to kinetic waveforms from participants running on a track across a range of velocities. We estimated GRF waveforms with three inertial sensors from participants running in a real-world training scenario: 400 m repeats at prescribed paces. Three specific findings can be summarized briefly here: (1) we estimated four-second GRF waveforms from the IMU data of the same duration, (2) estimations of contact time from the output waveform were accurate, but were overestimated at average running velocities >3.16 m s^−1^, and (3) estimates of single kinetic variables matched the overall trends of the measured input data, however the model tended to underestimate kinetic variables (stance average forces, peak force and average loading rate) at running velocities >3.16 m s^−1^ (Figures 3, 5). We have presented the baseline performance of these models using a LOOCV process and the transfer of the model for use on data from a novel participant.

The estimation of gait events from IMUs have been reported with a wide variety of algorithmic methods, as these are the most critical variables for parsing biomechanical waveforms (34–36). In the present study, IC difference between estimated and measured gait events ranged from 0.013–0.020 s across a range of running velocities. This temporal difference may have been due to the iterative corrections algorithm that was utilized to match estimated IC calculated from the output of the LSTM with measured IC. Estimation of TO differences ranged from −0.012–0.041 s. These differences appear to be specific to running velocity, as estimation of TO at velocities <3.16 m s^−1^ occurred before measured TO, and at running velocities >3.16 m s^−1^ TO was estimated to occur after measured TO. It follows that contact time was overestimated at velocities >3.35 m s^−1^. Previous work reported an RMSE of 0.011 s and *r*^2^ = 0.665 from a quantile regression forest (9). Our results show a threefold increase in the RMSE to 0.032 s but a stronger correlation *r*^2^ = 0.795. Greater error in our estimates likely came from greater variability in the average running velocity throughout a trial and the inclusion of accelerations and decelerations within a running trial.

Stance phase ground reaction force RMSE was comparable to ranges presented in previous work (RMSE of 0.39 BW) (10), with our estimated waveforms resulting in average RMSE of 0.245 BW for all running velocities. Another study reported an RMSE ranging from 0.13–0.17 BW between velocities of 2.7 and 4.5 m s^−1^ (22), using an algorithm that is closest in nature to ours, as they estimated portions of waveforms that could be concatenated into full GRF waveforms. The performance of our algorithm was similar to these previously reported values, with an RMSE ranging from 0.189–0.288 BW (Table 3), across a wider range of velocities and at non-constant velocities. The primary difference between our current study and previous work in this area is that previous studies estimated whole GRF waveforms in the laboratory at steady state running velocities on a treadmill, and only estimated stance phase or a segment of the waveform. Ours is the first study to produce a model for estimation of a full GRF waveform with multiple stance and swing phases from data collected outside of the laboratory.

In our study, measured stance average GRFs generally increased with velocity (Figure 3 Panel A), however not linearly as expected (29). Estimated stance average GRFs were underestimated when compared to measured stance average GRFs (Figures 2 Panel A, 5 Panel A). Divergence between estimated and measured values occurred over the same range of velocities (3.16 to 5.36 m s ^−1^) that contact time was overestimated (Figures 2, 5 Panel A). Generally, an increase in contact time will cause a decrease in stance average GRFs. This is compounded with the underestimation of peak GRF values at faster average running velocities (Figure 5 Panel C). Faster running velocities revealed a greater bias in estimated stance average GRFs. For comparison, the physical model developed by (37), presented an average RMSE ranging from 0.681–1.302 BW for running velocities from 2 to 5 m s^−1^. Regardless, our results show notable improvement on this work, with an RMSE for estimated stance average GRF ranging from 0.063 to 0.218 BW (Table 5).

Estimation of impulse is the most mathematically complex variable presented in this work and it also has the poorest agreement between estimated and measured values. Impulse was expected to decrease as velocity increased (16, 38), which matches our results. Estimated impulse from a quantile regression forest was reported to have a strong correlation (*r* = 0.974) and an RMSE of 0.004 BW*s for running velocities between 3.8 and 5.4 m s^−1^ (9). Our results differ, with a weak correlation of *r*^2^ = 0.385 and an average RMSE across velocities 0.044 BW*s. These differences can be related, in part, to the discussion of errors above for both contact time and stance average GRFs. Another key difference is the variation in experience levels among our participants when compared to highly trained Division 1 endurance athletes. Beyond these differences, impulse is highly susceptible to errors in both the estimation of contact time and GRF magnitude, both of which had detectable bias in the current model.

As expected, peak force increased with running velocity (Figure 3 Panel C). This measure has been related to estimation of external load while running (16, 38). Estimation of peak GRFs across the range of running velocities was the most accurate output from the current model. However, it should be noted, at faster running speeds the peak vertical ground reaction forces did not continue to increase; this may be a limitation of the mechanical function of the sensors (e.g., sampling frequency or physical limitations). Previous research reported that the relationship between peak GRFs estimated by an ANN at three different velocities (ranging from 2.78–3.89 m s^−1^) had a moderate correlation for peak GRF; *r*^2^ = 0.72 and 95% LoA [−0.17 0.18] BW, with a bias of 0.01 BW, on average (10). In contrast, our model had a slightly weaker correlation (*r*^2^ = 0.614) and LoA [0.322 0.381] BW, with an average RMSE of 0.223 BW. Although our model resulted in similar correlations, we also have twice as much variability represented by our 95% LoA range. Further investigation revealed an outlier from the peak GRF analysis, in which the value was overestimated by approximately 50% for a single participant. This observation indicates that the force-measuring insoles were moving between the foot and the shoe for this participant.

Measured loading rate generally increased with running velocity, as expected (Figure 3 Panel D) (38). Wouda et al. reported an ANN-estimated loading rate with correlation of *r*^2^ = 0.57, LoA of [−16 10] BW s^−1^ and a bias of −2.9 BW s^−1^ (10). Our results showed a correlation of *r*^2^ = 0.405, with LoA [−20.450 8.243] BW s^−1^ and a bias of −6.116 BW s^−1^, demonstrating less agreement and a larger bias than the previous work. This could be due in part to differences in data collection protocols and the sensitivity of the force sensing insoles to error in the calculation of loading rate (39). Estimated average loading rate was underestimated at velocities > 3.16 m s^−1^, possibly due to errors in the estimation of gait events. Identification of IC prior to the measured gait event decreases the estimated average loading rate. We attempted to mitigate this by estimating average loading rate between 10% and 40% contact time.

There are several limitations in this work. The force sensing insoles occasionally lost connection during trials, which led to different calibration files for the same participant. Force sensing insoles rely heavily on the calibration process prior to the data collection, and if they move between the foot and the shoe the force values will be affected. We noted this anecdotally from comments from a few participants that their shoes became slightly loose, which may have led to the insole moving slightly between the foot and the shoe. These sources of error likely contributed to the variability within our data and affected the machine learning model for the estimation of GRF waveforms. Given the IMUs were not perfectly rigidly attached to the user or embedded in the participants shoes or waistband, there is the potential for movement artifact of the IMU throughout the gait cycle. The methodology presented in this work is transferable to real-world running. However, we hesitate to recommend the algorithm in current form as a tool for the analysis of training and the translation of this work into the real-world environment. Overestimation of contact time with increased running velocity is an example of the limited transferability of the algorithm to novel environments. Building a machine learning model for a single participant or a small subset of participants with similar running ability would substantially reduce the model's estimation error. We have demonstrated a baseline for performance of a machine learning algorithm outside of the laboratory by presenting data from a LOOCV. We had a single participant run at the slowest velocities, and this participant's data did not follow the expected kinetic trends. However, this participant's data provide a good benchmark to demonstrate how these methods capture running performance of a truly novice runner. This work has improved upon much of the relevant literature for estimation of spatial-temporal and kinetic measures from the estimated ground reaction force waveforms. Future studies investigating the effects of differing volumes of data input, and potentially the inclusion of a wider range of running velocities should improve estimations from similar machine learning algorithms.

In conclusion, the mapping of GRF waveforms from IMU data collected in a real-world environment has been shown to be feasible, with limitations. We have presented conservative results from an LSTM model of GRF waveform estimation by reporting data from a LOOCV analysis. We used three IMUs for the mapping of inertial to kinetic data for a variety of participants ranging in skill from truly novice runner (30:00 estimated 5 km race time) to more highly trained runners (15:30 5 km race time) running 400 m on a square track. Additionally, it would be valuable to identify biases in the reported variables by comparing measurement of force data from a force-instrumented treadmill to those measured by force sensing insoles, across a range of velocities and inclinations matching the training environment of experienced runners.

## Data Availability

The raw data supporting the conclusions of this article will be made available by the authors, without undue reservation.
